# Feasibility and accuracy of coronary artery calcium score on virtual non-contrast images derived from a dual-layer spectral detector CT: A retrospective multicenter study

**DOI:** 10.3389/fcvm.2023.1114058

**Published:** 2023-03-02

**Authors:** Panpan Yang, Ren Zhao, Wei Deng, Shutian An, Yuguo Li, Mao Sheng, Xingbiao Chen, Yingfeng Qian, Yongqiang Yu, Dan Mu, Yining Wang, Xiaohu Li

**Affiliations:** ^1^Department of Radiology, The First Affiliated Hospital of Anhui Medical University, Hefei, Anhui, China; ^2^Research Center of Clinical Medical Imaging, Anhui Province Clinical Image Quality Control Center, Hefei, Anhui, China; ^3^Department of Cardiology, The First Affiliated Hospital of Anhui Medical University, Hefei, Anhui, China; ^4^Department of Radiology, The Second People's Hospital of Hefei, Hefei Hospital Affiliated to Anhui Medical University, Hefei, Anhui, China; ^5^Clinical Science, Philips Healthcare, Shanghai, China; ^6^Department of Radiology, Affiliated Nanjing Drum Tower Hospital of Nanjing University Medical School, Nanjing, China; ^7^Department of Radiology, Peking Union Medical College Hospital, Beijing, China

**Keywords:** coronary artery calcium score, coronary artery disease, computed tomography angiography, dual energy CT (DECT), dual energy CT system

## Abstract

**Rationale and objective:**

This retrospective study was to evaluate the feasibility and accuracy of coronary artery calcium score (CACS) from virtual non-contrast (VNC) images in comparison with that from true non-contrast (TNC) images.

**Materials and methods:**

A total of 540 patients with suspected of coronary artery disease (CAD) who underwent a dual-layer spectral detector CT (SDCT) in three hospitals were eligible for this study and 233 patients were retrospectively enrolled for further analysis. The CACS was calculated from both TNC and VNC images and compared. Linear regression analysis of the CACS was performed between TNC and VNC images.

**Results:**

The correlation of overall CACS from VNC and TNC images was very strong (*r* = 0.923, *p* < 0.001). The CACS from VNC images were lower than that from TNC images (221 versus. 69, *p* < 0.001). When the regression equation of the overall coronary artery was applied, the mean calibrated CACS-VNC was 221 which had a significant difference from the CACS-TNC (*p* = 0.017). When the regression equation of each coronary branch artery was applied, the mean calibrated CACS-VNC was 221, which had a significant difference from the CACS-TNC (*p* = 0.003). But the mean difference between the CACS-TNC and the calibrated CACS-VNC in either way was less than 1. The agreement on risk stratification with CACS-TNC and CCACS-VNC was almost perfect.

**Conclusion:**

This multicenter study with dual-layer spectral detector CT showed that it was feasible to calculate CACS from the VNC images derived from the spectral coronary artery CT angiography scan, and the results were in good accordance with the TNC images after correction. Therefore, the TNC scan could be omitted, reducing the radiation dose to patients and saving examination time while using dual-layer spectral detector CT.

## Introduction

Coronary artery disease (CAD) is the most common type of cardiovascular diseases globally and the leading cause of death (1). Coronary artery calcium score (CACS) is a quantitative method to determine the calcium buildup on the wall of coronary artery from CT imaging. The CACS was effectively linked to cardiovascular risks across ethnic groups, regardless of age, sex and risk factors (2, 3) to predict future cardiovascular events. Previous study showed that an extremely high CACS (≥1,000) was associated with increased risks of coronary heart disease, other cardiovascular diseases, cancers, and all-cause mortalities (4). The CACS was also useful in deciding initiation or continuation of pharmacological and lifestyle therapies to prevent cardiovascular diseases (5).

A routine non-contrast scan was often taken before the coronary artery CT angiography (CCTA) scan to obtain a CACS (6). The CCTA is widely used non-invasively to rule out coronary artery stenosis in patients with suspected CAD (7). Because of the contrast medium containing iodine, the CT value of calcified plaque is similar to that of the contrast medium, and therefore, it is difficult to quantify the calcified plaque by CCTA, but it can be used to identify non-calcified plaque (8). Dual-energy or spectral CT is an emerging technology that enables identification of different materials (e. g. iodine) using a material decomposition algorithm based on high-and low-energy X-ray attenuation (9). Therefore, virtual non-contrast imaging (VNC) is an image post-processing technique used to create ‘non-contrast’ images of contrast-enhanced scans via the subtraction of iodine. The dual-layer spectral detector CT (SDCT) is the latest detector-based imaging method. Compared with other tube-based (rapid tube voltage switch or dual-source) dual-energy CT, it can provide exactly matched high-and low-energy X-ray attenuation, apply material decomposition algorithm in projection domain and not require pre-selection of the scan protocol (10). Some previous studies demonstrated that the VNC images derived from multi-phase contrast scan can replace the non-contrast scan (TNC) (11–13).

Further, several studies demonstrated that the VNC images generated from spectral CCTA data could be used for CACS calculation (12, 14–16). Gassert et al. showed that the CACS calculated from the VNC images were highly consistent with that from TNC images (13). Nevertheless, many of the previous studies were single center, and only a few studies further compared the impact on cardiovascular risk stratification while using CACS from the VNC images. Using the VNC images to calculate CACS can omit the TNC scan procedure and reduce radiation dose to patients. Therefore, the purpose of this multi-center study was to investigate the accuracy of CACS from the VNC images compared with the TNC images and validate the clinical feasibility.

## Materials and methods

### Study population and radiation dose

**Figure 1:**
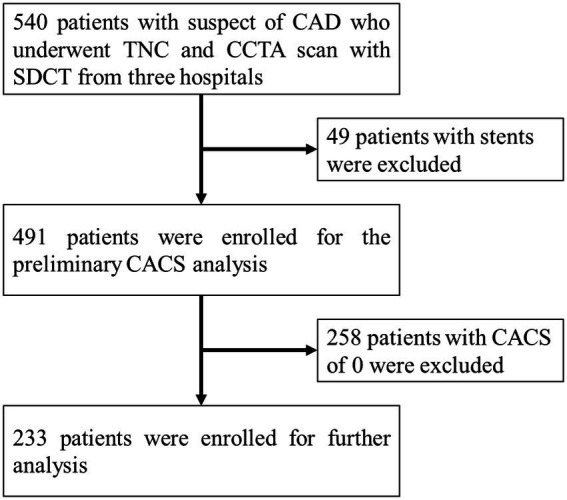
Study flowchart for the patient selection. The CAD represents coronary artery disease, the TNC represents non-contrast images, the CCTA represents coronary artery CT angiography, the SDCT represents dual-layer spectral detector CT, and the CACS represents coronary artery calcium score.

A total of 540 patients with suspected CAD who underwent TNC and CCTA scans using SDCT from June 2018 to July 2021, in three hospitals (Peking Union Medical College Hospital, The Second Peoples Hospital of Hefei and Nanjing Drum Tower Hospital) were retrospectively included for the analyzes. Among them, 49 patients with coronary stents and 258 patients with a CACS of 0 calculated from the TNC images were excluded after preliminary image analysis. Then, a total of 233 patients were included for further analyzes (Figure 1). Among the 233 patients, 95 patients were from Peking Union Medical College Hospital, 88 patients were from The Second Peoples Hospital of Hefei, and 50 patients were from Nanjing Drum Tower Hospital. The volume computed tomography dose index (CTDI_vol_) and dose length product (DLP) of TNC and CCTA scans were analyzed retrospectively. The effective dose (ED) was calculated using the formula: ED (mSv) = DLP × k, where k was the chest (heart) effective dose conversion factor, k = 0.014 mSv•mGy^−1^_•_cm^−1^ (17).

### Imaging protocol

All patients underwent TNC and CCTA ECG-gated scans in the three centers using the SDCT scanner (IQon Spectral CT; Philips Healthcare, Best, The Netherlands). The main scan parameters of the three centers for TNC were: tube voltage of 120 kVp, tube current ranging from 62 to 191 mA with dose modulation enabled, gantry rotation time of 0.33 s, collimation of 64×0.625 mm, FOV ranging from 206 to 273, reconstruction filter of CB, slice thickness of 2.5 mm, matrix of 512×512. A dose of 50–60 mL of iodine contrast agent was injected at a flow rate of 4–5 mL/s followed by 40–50 mL of saline with same rate. The bolus tracking technique was applied with a trigger threshold of 100 HU in the ascending aorta for the CCTA scan. The scan parameters for CCTA were: tube voltage of 120 kVp, tube current ranging from 147 to 752 mA with dose modulation enabled, gantry rotation time of 0.27 s, collimation of 64×0.625 mm, pitch of 0.16, FOV ranging from 206 to 273, reconstruction filter of CB, slice thickness of 0.9 mm with increment of 0.45 mm, matrix of 512×512. Conventional images were reconstructed using the iterative reconstruction algorithm (iDose-3), and spectral-based-images (SBI) were reconstructed using a spectral algorithm (level 3). For CCTA imaging, Iodinated contrast: Peking Union Medical College Hospital (Iopamiro,370 mgI/mL,Bracco Sine Pharma); The Second Peoples Hospital of Hefei (iodixanol,320 mg/mL,GE Healthcare); Nanjing Drum Tower Hospital (iopromide,370 mg/mL,Ultravist, Bayer).

### Post-processing and analysis of image

#### Coronary artery calcium score

**Figure 2:**
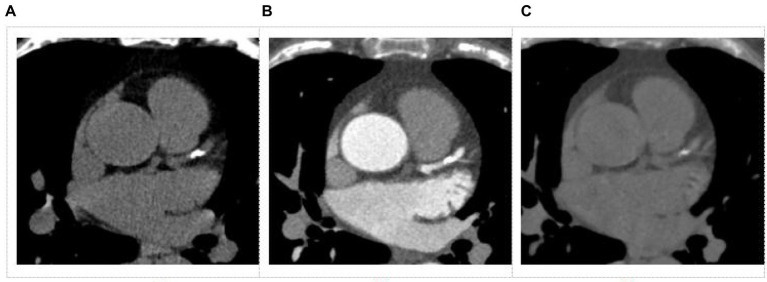
Images obtained from a dual-layer spectral detector CT for coronary artery calcium score. **(A)** Conventional non-contrast image, **(B)** Coronary artery CT angiography (CCTA) image, **(C)** Virtual non-contrast image from a CCTA image.

The conventional images and SBI data were transferred onto a dedicated workstation (IntelliSpace Portal 10; Philips Healthcare) for post-processing and further analysis. The VNC images were derived from CCTA SBI data with slice thickness setting of 2.5 mm which was same as TNC. Representative samples of TNC and VNC images from the same patient are shown in Figure 2. Images were evaluated blindly by two experienced radiologists (with more than 5 years of working experience in cardiovascular radiology). The CACS was calculated using a semi-automatic software HBCS (Heartbeat calcium scoring, Philips Healthcare) by the Agatston method (18). The CACS calculated from the TNC images were used as references. The total CACS of the coronary artery were calculated from VNC and TNC images. And the CACS of each branch of the coronary artery (LM, LAD, LCX, and RCA) were also determined. Linear regression analysis was performed between the CACS of the overall coronary artery or each branch artery from the TNC and VNC images. The branch arteries with CACS of 0 were excluded while performing regression analysis. Two ways were used to correct the CACS from the VNC: 1) the regression equation was derived from the overall coronary artery and applied to each branch artery of every patient; 2) the regression equation was derived from each branch artery and applied to the corresponding branch artery of every patient, and the sum from branch arteries was recorded as CACS of overall coronary artery. The corrected CACS from the VNC images was recorded as CCACS-VNC. Cardiovascular risk stratification was performed using CACS-TNC as a reference. And the cardiovascular risk was re-stratified using CCACS-VNC (two ways) consecutively. The following categories were used for risk classification (19): 0 (no risk), 1–100 (low to moderate risk), 101–400 (moderate risk), and more than 401 (high risk).

### Statistical analysis

SPSS version 26.0 (IBM Corp, Armonk, NY, USA) statistical software was used for statistical analysis. Continuous variables were expressed as mean ± standard deviation (SD) and categorical variables were expressed as frequencies or percentages. The Kolmogorov–Smirnov test was used for testing normality of continuous variables. The correlation of the CACS between the TNC and VNC images were determined by Spearman correlation coefficient. Additionally, the Kruskal-Wallis H test was performed to compare the difference between CACS from TNC images from three centers. The agreement was defined as negligible correlation (0.00 < *r* < 0.10), weak correlation (0.10 < *r* < 0.39), moderate correlation (0.40 < *r* < 0.69), strong correlation (0.70 < *r* < 0.89), and very strong correlation (0.90 < *r* < 1.00) (20, 21). After the regression equation was applied, the CCACS-VNC was compared with CACS-TNC using Wilcoxon signed rank sum test. Additionally, the CCACS-VNC that was calculated by two ways were also compared using Wilcoxon signed rank sum test. Bland–Altman plot was constructed to evaluate the consistency between the CACS-TNC and CCACS-VNC. The SD and SNR of the ascending aorta from TNC and VNC images were also compared by the Wilcoxon signed rank sum test. Cohen Kappa test was used to evaluate the agreement of cardiovascular risk stratification results from CACS-TNC and CCACS-VNC. The agreement was defined as slight agreement (0 < Kappa value ≤0.2), fair agreement (0.2 < Kappa value ≤0.4), moderate agreement (0.4 < Kappa value ≤0.6), substantial agreement (0.6 < Kappa value ≤0.8), and almost perfect agreement (0.8 < Kappa value ≤1.0) (22). The G*Power was used to calculate the study power. A *p* value of <0.05 was considered statistically significant.

## Results

### Characteristics of study population and radiation dose

A total of 233 patients (129 men, mean age of 63 ± 10 years) with CACS of >0 were included for further analysis. While for risk stratification analysis, additional 258 patients with CACS of 0 included. The CTDI_vol_ was 3.9 ± 1.2 mGy•cm, and the ED was 0.7 ± 0.3 mSv for the TNC scan; The CTDI_vol_ was 38.7 ± 16.8 mGy•cm, the ED was 8.0 ± 3.5 mSv for the CCTA scan. Details of the study population and radiation dose are shown in Table 1.

**Table 1:** Baseline characteristics of patients.

Cardiovascular risk factors	
Age	63 ± 10 years
Male gender (%)	129 (55.3%)
BMI (Body Mass Index)	27 ± 3 kg/m^2^
Arterial hypertension (%)	135 (57.7%)
Hypercholesterolemia (%)	13 (5.5%)
Diabetes (%)	61 (26.1%)
Smoker (%)	18 (7.7%)
CT radiation dose	
TNC CTDI_vol_, mGy•cm	3.9 ± 1.2
TNC ED, mSv	0.7 ± 0.3
CCTA CTDI_vol_, mGy•cm	38.7 ± 16.8
CCTA ED, mSv	8.0 ± 3.5

TNC represents conventional non-contrast. VNC represents virtual non-contrast. CTDI_vol_ represents volume computed tomography dose index. ED represents effective dose. Values are mean ± SD, median (inter-quartile range), or *n* (%).

### Comparison of calcium score between the true non-contrast-enhanced images and the virtual non-contrast-enhanced images

**Figure 3:**
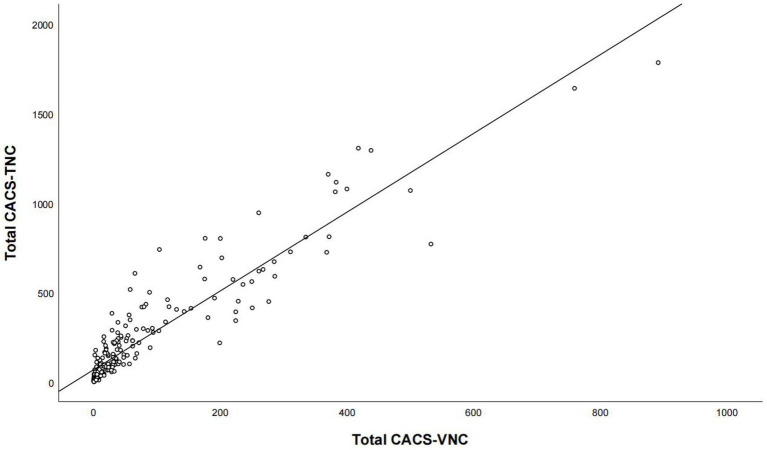
The correlation analysis for the overall calcium score between the conventional non-contrast and virtual non-contrast images. Total CACS-VNC represents the overall coronary artery calcium score from the virtual non-contrast images, and total CACS-TNC represents the overall coronary artery calcium score from the non-contrast images.

For the overall coronary artery, CACS-TNC was significantly higher than CACS-VNC (221 versus. 69, *p* < 0.001), but with a very strong positive correlation (*r* = 0.923, p < 0.001, Figure 3). For coronary artery branches (LM, LAD, LCX, RCA), the correlations were strong between the CACS-TNC and CACS-VNC with r values of 0.835, 0.871, 0.854, and 0.842 (all *p* < 0.001) respectively. The detailed results are presented in Table 2. No significant differences in CACS-TNC among the three centers were seen (*p* = 0.274, respectively).

**Table 2:** Comparison of CACS from the TNC and VNC images.

	Total	LM	LAD	LCX	RCA
CACS-TNC	221 ± 295	96 ± 123	132 ± 141	83 ± 100	107 ± 154
CACS-VNC	69 ± 125	36 ± 67	44 ± 72	22 ± 35	29 ± 65
*r* value	0.923	0.835	0.871	0.854	0.842
Value of *p*	<0.001				

The CACS-TNC represents the coronary artery calcium scores from the conventional non-contrast images. The CACS-VNC represents the coronary artery calcium scores from the virtual non-contrast images. The CACS-VNC represents the coronary artery calcium volume from virtual non-contrast images. Total, the overall coronary artery; LM, left main coronary artery; LAD, left anterior descending branch; LCX, left circumflex branch; RCA, right coronary artery. Values are mean ± SD.

### Calibrated calcium score from virtual non-contrast-enhanced images

The regression equation for the overall coronary artery was Y = 68.48 + 2.2*X. After the regression equation was applied, the mean CCACS-VNC was 221 which had a significant difference from CACS-TNC (*p* = 0.017). But the difference was less than 1 (p < 0.001). The overall regression equation was also applied to calibrate each coronary artery branch calcium score and compared with the CACS-TNC. The detailed results were listed in Table 3.

**Table 3:** Comparison of CACS-TNC, CCACS-VNC_AVG_, and CCACS-VNC_branches_.

	CACS-TNC	CCACS-VNC_AVG_	CCACS-VNC_branches_	Value of *p*
Total	221 ± 295	221 ± 275^*^	221 ± 254^*^	<0.001
LM	96 ± 123	147 ± 147^*#^	96 ± 105	<0.001
LAD	132 ± 141	166 ± 158^*#^	132 ± 122	<0.001
LCX	83 ± 100	117 ± 76^*#^	83 ± 80^*^	<0.001
RCA	107 ± 154	132 ± 143^*#^	107 ± 131^*^	<0.001

The CACS-TNC represented the coronary artery calcium scores from the conventional non-contrast images; The CCACS-VNC_AVG_ represented the calcium scores after correction using the regression equation of the overall coronary artery; The CCACS-VNC_branches_ represented the sum of CACS corrected using the corresponding regression equation of each branch artery; The CACS-TNC represented as the reference standard. ^*^Compared with the CACS-TNC, *P* < 0.05; ^#^compared with CCACS-VNC_branches_, *p* < 0.05. Total, the overall coronary artery; LM, left main coronary artery; LAD, left anterior descending branch; LCX, left circumflex branch; RCA = right coronary artery. Values are mean ± SD.

**Figure 4:**
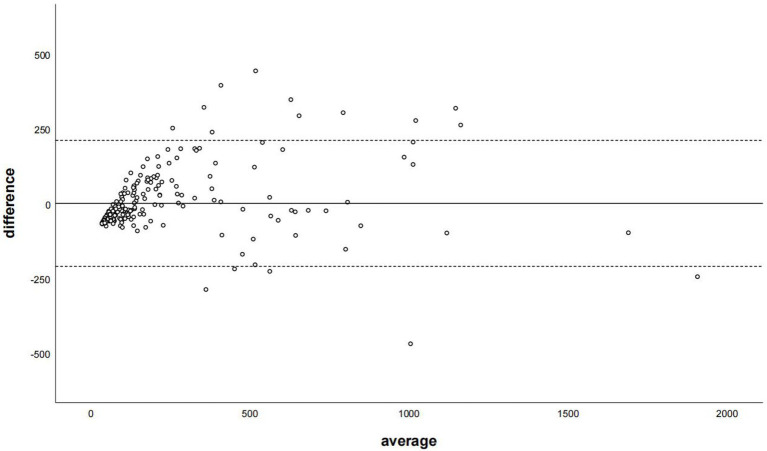
The Bland–Altman plot was used to describe the agreement of coronary artery calcium score between the non-contrast images and virtual non-contrast images corrected by the regression equation of the overall calcium score. The average represented the mean value of calcium score from two images, and the difference represented the different value of calcium score from two images.

**Figure 5:**
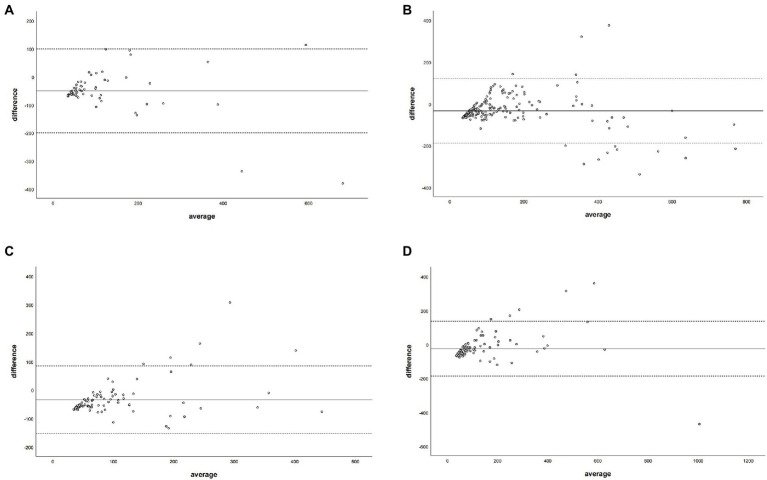
The Bland–Altman plot was used to describe the agreement of coronary artery calcium score between the non-contrast images and virtual non-contrast images (corrected by the regression equation of the overall calcium score) of each coronary artery branch. The average represented the mean value of calcium score from two images, and the difference represented the different values of calcium score from two images. Panel **(A)** denotes LM = left main coronary artery, panel **(B)** depicts LAD = left anterior descending branch, panel **(C)** depicts LCX = left circumflex branch, and panel **(D)** depicts RCA = right coronary artery.

The Bland–Altman plot determined that the mean difference of CACS between the CACS-TNC and CCACS-VNC was 0.03 (95% CI: −210.66, 210.73; Figure 4); the mean difference for each coronary artery branch (LM, LAD, LCX, and RCA) were − 50.33, −33.85, −34.51, and − 25.39 (Figure 5).

**Figure 6:**
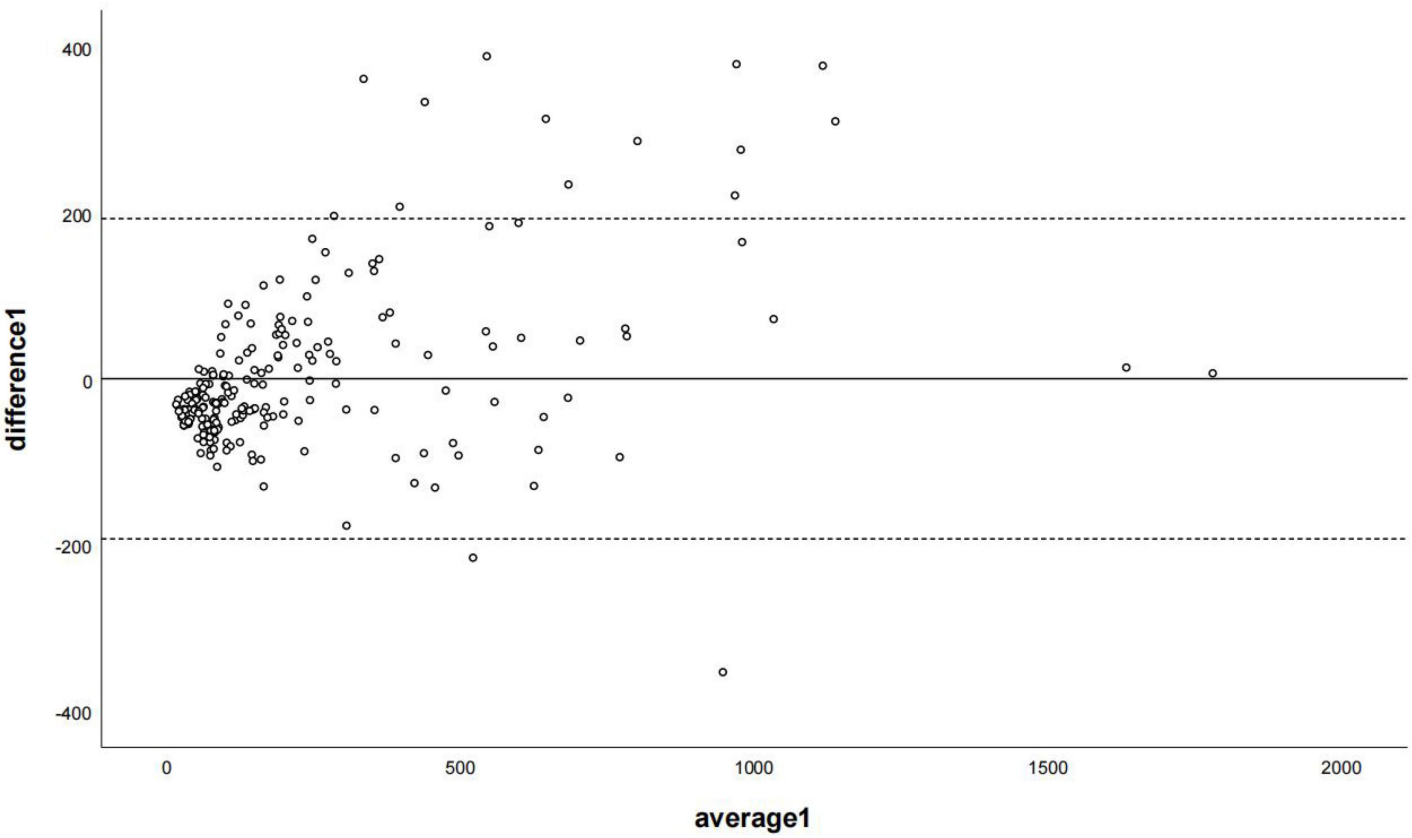
The Bland–Altman plot was used to describe the agreement of coronary artery calcium scores between the non-contrast images and virtual non-contrast images (the sum of calcium scores was corrected using the corresponding linear regression equation of each coronary branch). Average 1 represented the mean value of calcium score from two images, and difference 1 represented the different values of calcium score from two images.

**Figure 7:**
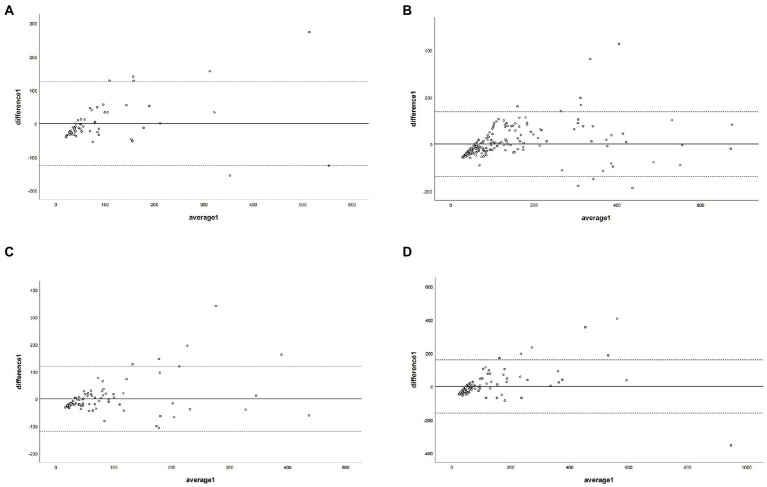
The Bland–Altman plot was used to describe the agreement of coronary artery calcium score between the non-contrast images and virtual non-contrast images (corrected by the corresponding regression equation of each coronary branch). Average 1 represented the mean value of calcium scores from two images, and difference1 represented the different values of calcium score from two images. Panel **(A)** depicts LM = left main coronary artery, panel **(B)** depicts LAD = left anterior descending branch, panel **(C)** depicts LCX = left circumflex branch, and panel **(D)** depicts RCA = right coronary artery.

The regression equations for all branch arteries, LM, LAD, LCX, and RCA were *Y* = 40.22 + 1.58*X (LM), *Y* = 56.76 + 1.7*X(LAD), *Y* = 31.62 + 2.31*X (LCX), and *Y* = 48.4 + 2.02*X (RCA), respectively. After correction with the corresponding coronary artery branch regression equation, the CCACS-VNC for LM, LAD, LCX, and RCA were 96 ± 105,132 ± 122, 83 ± 80, and 107 ± 131, respectively. The CCACS-VNC of LM and LAD arteries had no significant difference with the CACS-TNC (both *p* > 0.05). The CCACS-VNC of LCX and RCA arteries had significant differences from the CACS-TNC (both *p* < 0.05). The sum of CCACS-VNC from all the branches was 221 ± 254, which was significantly different with CACS-TNC (*p* = 0.003), but the difference was less than 1 (*p* < 0.001). The detailed results are listed in Table 3. The Bland–Altman plot determined that the mean difference of total CACS between the CACS-TNC and CCACS-VNC was 0 (95% CI: −193.06, 193.01; Figure 6); the mean difference for each coronary artery branch (LM, LAD, LCX, and RCA) were 0.00, 0.07, −0.08, and − 0.11 (Figure 7).

Additionally, no significant statistical difference was found for CCACS-VNC of overall coronary artery calculated by two ways (using overall regression and branch regression) (*p* = 0.553); but the CCACS-VNC of all branch arteries showed significant differences (all *p* < 0.05). The detailed results are listed in Table 3.

### Cardiovascular risk stratification

A total of 491 cases, including 258 cases with a CACS of 0 and 233 cases with CACS greater than 0, were used for risk stratification. The CACS calculated from the VNC images were all 0 for the patients with CACS of 0 from the TNC images. Based on the CCACS-VNC calculated from the linear regression equation for the overall coronary artery, 46 cases (9.4%) were regrouped to the adjacent category compared with the results using the CACS-TNC (Table 4). The agreement of the risk stratification was almost perfect (Kappa value = 0.853). Based on the CCACS-VNC calculated from linear regression equation of each branch artery, 59 cases (12.0%) on the VNC images were regrouped to the adjacent category compared with the results using CACS-TNC (Table 5). The agreement of the risk stratification was almost perfect (Kappa value = 0.813).

**Table 4:** Comparison of the Agatston calcium scoring risk rating between the CACS-TNC and CCACS-VNC_AVG_.

	CCACS-VNC_AVG_					
CACS-TNC		0	1–100	101–400	>400	Total
	0	258	0	0	0	258
	1–100	0	95	22	0	117
	101–400	0	10	60	4	74
	>400	0	0	10	32	42
	Total	258	105	92	36	491

The CACS-TNC represented the coronary artery calcium scores from the conventional non-contrast images, the CCACS-VNC_AVG_ represented the calcium scores after correction using the regression equation of the overall coronary artery. In TNC and VNC images, there were 258 cases in which CACS was 0, which were not corrected by the linear regression equation. Those with CACS other than 0 in the VNC images were corrected by the linear regression equation (*Y* = 68.48 + 2.2*×) of the overall coronary artery. The total number of patients (491) referred to 258 patients with a CACS of 0 and 233 patients were included for further analysis.

**Table 5:** Comparison of the Agatston calcium scoring risk rating between the CACS-TNC and CCACS-VNC_branches_.

	CCACS-VNC_branches_					
CACS-TNC		0	1–100	101–400	>400	Total
	0	258	0	0	0	258
	1–100	0	78	39	0	117
	101–400	0	6	64	4	74
	>400	0	0	10	32	42
	Total	258	84	113	36	491

The CACS-TNC represented the coronary artery calcium scores from the conventional non-contrast images and the CCACS-VNC_branches_ represented the sum of CACS corrected using the corresponding linear regression equation of each coronary branch. Total represented the overall coronary artery calcium score. In the TNC and VNC images, there were 258 patients whose CACS was 0, which were not corrected by the linear regression equation. Those with the CACS other than 0 in the TNC images of each coronary artery branch were corrected by the corresponding linear regression equation of each coronary branch. The total number of patients (491) referred to 258 patients with a CACS of zero and 233 patients were included in the group.

### Image quality

The mean attenuation value of the ascending aorta in CCTA images was 405.93 ± 70.21 HU, and the mean attenuation value of the ascending aorta on the VNC images was slightly higher than that on the TNC images (43.98 ± 6.67 HU versus. 43.09 ± 4.33 HU, *p* = 0.023). And the difference in attenuation value of ascending aorta between the TNC and VNC images was less than 1 HU (*p* < 0.001). Image noise from the VNC images was lower than that from the TNC images (12.6 versus. 20.6, p < 0.001). The statistical power of this study was 0.99.

## Discussion

This retrospective multi-center study with large cohort patients demonstrated that the CACS derived from the VNC images of CCTA scan had a very strong correlation (*r* = 0.923) with the CACS derived from TNC scan. After the regression equation was applied, the corrected CACS from VNC was similar to the CACS-TNC (mean difference of <1). The agreement of risk stratification using the CACS-TNC and CCACS-VNC was almost perfect (kappa value of 0.853). Therefore, the TNC scan before CCTA can be omitted to reduce radiation dose to patients and save examination time.

The virtual non-contrast image was a basic feature of dual-energy CT or spectral CT, which could identify the contrast media and remove its contribution to the X-ray attenuation from contrast-enhanced images (23). Previous studies showed that the VNC images could replace the TNC images for the abdomen using SDCT (24, 25). Our study on the ascending aorta also showed similar results that the difference between TNC and VNC images was less than 1 HU. However, the CT attenuation value from the VNC images of calcium plaque was lower than that from the TNC images (221 versus. 69). The reason might have been that plaque with CT attenuation greater than 130HU was recognized as calcium plaque during CACS calculation. The calcium plaque consisted of a complex mixture because calcification is a complex, organized, regulated, and active process (26). The pattern of some parts of the plaque might have been more iodine-like for the spectral material decomposition algorithm. Thus, these parts might have been identified as iodine contrast media and removed, resulting in lower CT attenuation of calcium plaque in the VNC images. Further, because 130 HU was kept as the same to differentiate calcium and non-calcium plaque for both TNC and VNC images, the underestimation of CT attenuation for calcium plaque could have caused smaller calcium volume from the VNC images (186 versus. 71). Consequently, CACS from the VNC images might have been much lower than that from the TNC images according to the Agatston score method.

Although the CACS difference was significant between the TNC and VNC images, the correlation between them was very strong, which was in line with the results of previous studies (12, 13). Using the linear regression analysis, the slope of the regression line determined by Gassert et al. was 3.83 (13), and the slope was 2.3 from the study conducted by Nadjiri et al. The slope of the regression line in this study was 2.2, which was closer to the results from Nadjiri et al. (12). The regression equation in this study was used to correct the CACS-VNC, which was different from the previous study. Besides the CACS of the overall coronary artery, we also performed regression analysis for each branch artery with a slope from 1.58 to 2.31. The CCACS-VNC calculated in two ways (using overall regression and branch regression) had no significant difference for the overall coronary artery; the difference was less than 1 for CCACS-VNC in both ways, compared to CACS-TNC. But for the branch arteries (LM, LAD, LCX, and RCA), the differences in CCACS-VNC calculated by the overall regression way in comparison with CACS-TNC were − 50.33, −33.85, −34.51, and − 25.39, while they were 0.00, 0.07, −0.08, and − 0.11 using branch regression way. The branch regression way could provide a more accurate result for the branch artery follow-up. However, a single slope or regression equation was more reasonable for daily practice. Thus, further study is needed to validate which way is better.

All the scan parameters among three centers were the same except some variations in tube current, and the agreement of risk stratification between TNC and VNC images was almost perfect. While using the overall regression, 445 (90.6%) patients were assigned to the same category; 432 (88.0%) patients were with branch regression way. Our results were comparable with the outcomes of previous studies conducted by Gassert et al. (83.3%) and Dan Mu et al. (93%). Although Mu et al. proposed a deep learning method to calculate CACS from the CCTA images (27), the method required further clinical validation. The software (HBCS) used in this study is commercially available and the Agatston score method has been widely used to identify biomarkers for cardiovascular risk stratification for over three decades (28).

## Limitations

There were several limitations in our study. First, the reference standard in this study was the TNC image, which was more likely affected by beam hardening artifacts or calcium blooming artifacts. Second, only the Agatston method was used to calculate the CACS, and other methods such as mass integral or volume integral methods were not used in this study. Second, the contrast medium injection protocols in this retrospective study were not the same, which might impact the VNC results. Although previous studies using different contrast medium injection protocols (12, 13, 27), similar conclusions were presented. Some prospective studies shall be performed to investigate the impact on the accuracy of VNC results caused by contrast medium injection protocols.

## Conclusion

In conclusion, this multicenter study with dual-layer spectral detector CT showed that it was feasible to calculate CACS from the VNC images derived from the spectral coronary artery CT angiography scan, and the results were in good accordance with the TNC images after correction. Therefore, the TNC scan could be omitted, reducing the radiation dose to patients and saving examination time while using dual-layer spectral detector CT.

## Data availability statement

The datasets used and analyzed during the current study are available from the corresponding author on reasonable request. Requests to access the datasets should be directed to zhaorenlxh@126.com.

## Ethics statement

The studies involving human participants were reviewed and approved by The First Affiliated Hospital of Anhui Medical University. The ethics committee waived the requirement of written informed consent for participation.

## Author contributions

All authors listed have made a substantial, direct, and intellectual contribution to the work and approved it for publication.

## Funding

This work was supported in part by the National Natural Science Foundation of China (82071897 and 81970446) and National Science Foundation for Distinguished Young and Scholars of the Higher Education Institutions of Anhui Province, China (2022AH020071).

## Conflict of interest

The authors declare that the research was conducted in the absence of any commercial or financial relationships that could be construed as a potential conflict of interest.

## Publisher’s note

All claims expressed in this article are solely those of the authors and do not necessarily represent those of their affiliated organizations, or those of the publisher, the editors and the reviewers. Any product that may be evaluated in this article, or claim that may be made by its manufacturer, is not guaranteed or endorsed by the publisher.
